# *Gentianella acuta* mitigates cardiovascular damage and inflammation in diet-induced hypercholesterolaemic rats

**DOI:** 10.3892/etm.2021.10694

**Published:** 2021-09-06

**Authors:** Mingdong Si, Meng Wu, Yingying Huo, Aiying Li, Shengjiang Guan, Donglai Ma, Zhihong Ma

**Affiliations:** 1 Department of Traditional Chinese Medicine, School of Pharmacy, Hebei University of Chinese Medicine, Shijiazhuang, Hebei 050200, P.R. China; 2 Department of Traditional Chinese Medicine, College of Pharmaceutical Sciences, Yunnan University of Traditional Chinese Medicine, Kunming, Yunnan 650000, P.R. China; 3 Hebei Higher Education Institute Applied Technology Research Center on TCM Formula Preparation, Shijiazhuang, Hebei 050091, P.R. China; 4 Department of Immunology, School of Basic Medicine, Hebei University of Chinese Medicine, Shijiazhuang, Hebei 050200, P.R. China; 5 Traditional Chinese Medicine Processing Technology Innovation Center of Hebei Province, Shijiazhuang, Hebei 050200, P.R. China

**Keywords:** *Gentianella acuta*, hypercholesterolaemia, cardiovascular disease, IKK/IκB/NF-κB

## Abstract

*Gentianella acuta* (*G. acuta*) has been widely used as a traditional medicine by Chinese Mongolian populations for the treatment of heart diseases and has also been tested in modern pharmacological experiments. However, the effects of *G. acuta* on cardiovascular damage and inflammation under conditions of hypercholesterolaemia remain unclear. The present study investigated the effects and mechanisms of the water extract of *G. acuta* on cardiovascular damage and inflammation caused by a high-cholesterol diet. Male Sprague-Dawley rats were fed a high-cholesterol diet for 4 weeks to establish the hypercholesterolaemia rat model, and they were administered physiological saline or 1.2 g/kg of *G. acuta* by gavage starting from the 15th day. After the last administration, the blood, heart and thoracic aorta samples were collected and examined. It was revealed that *G. acuta* treatment could ameliorate cardiomyocyte disorder and thoracic aortic vessel wall damage, reduce serum lipid levels and inflammatory factors and improve heart function. Compared with the Model group, the serum levels of triglycerides, total cholesterol, low-density lipoprotein and tumour necrosis factor-α were decreased, and the high-density lipoprotein and interleukin-10 levels were increased in the Model-G group. Moreover, in both the heart and thoracic aorta, *G*. *acuta* reduced the expression and phosphorylation of inhibitor of nuclear factor kappa-B kinase β (IKKβ), inhibitor of NF-κB-α (IκBα) and p-nuclear factor kappa-B (NF-κB). Therefore, *G*. *acuta* may exert an inhibitory effect on the IKK/IκB/NF-κB signalling pathway to protect the heart and thoracic aorta in hypercholesterolaemic rats.

## Introduction

Cardiovascular disease (CVD) is an important health concern and has been the focus of considerable research. In China, there are an estimated 330 million patients with CVD, and CVD accounts for >40% of all disease-related resident deaths and has been identified as the leading cause of mortality (1). The mortality, incidence and prevalence rates of CVD continue to increase globally (1). Atherosclerosis (AS) is a major cause of CVD and the result of several factors, among which lipid metabolism disorders are the leading contributor. In modern society, lipid metabolism disorders are caused by hypercholesterolaemia and induce an inflammatory response that is involved in all processes of AS (2,3). The nuclear transcription factor controlling their release is phosphorylated (p)-nuclear factor κB (NF-κB), which has been revealed to induce an increase in the production of inflammatory and adhesion factors (4-6). Usually, NF-κB and the inhibitory protein inhibitor of NF-κB-α (IκBα) exist as a complex and are inactive. However, when cells are stimulated or activated, the inhibitor of NF-κB kinase β (IKKβ) phosphorylates and degrades IκBα, thus activating NF-κB. p-NF-κB translocates into the nucleus, binds to related genes and regulates their transcription (7-10). In addition, numerous studies have reported that oxidative stress is an important cause of AS and is closely associated with NF-κB (11-13). Therefore, the occurrence of AS is closely linked to the IKK/IκB/NF-κB pathway.

*Gentianella acuta* (Michx.) Hulten (*G. acuta*) belongs to the *Gentianella* genus of the Gentianaceae family, also known as the bitter gentian (14). The Elunchun people have been using *G. acuta* to treat arrhythmias and other heart diseases for thousands of years (15,16). Previous studies on *G. acuta* mainly have focused on traditional efficacy, such as liver protection, and anti-arrhythmic, antioxidant and hypoglycaemic effects; however, further discoveries have been made in other fields (17); for example, the bioactive substances of *G. acuta* have been revealed to exert a beneficial effect on aberrant intestinal motility (18-20). Li *et al* (18) reported treatment with water extract of *G. acuta* could ameliorate cardiac structural disorders, excessive collagenous fiber accumulation in the heart and cardiac malfunction by regulating the NF-κB pathway in a model of myocardial fibrosis. Wang *et al* (21) indicated that xanthones from *G. acuta* exerted cardioprotective effects on myocardial ischemia/reperfusion (I/R) injury through its antioxidant and anti-apoptosis properties. Yang *et al* (22) indicated that the aqueous extract of *G. acuta* may improve isoproterenol-induced myocardial fibrosis through the inhibition of the tumour growth factor (TGF)-β1/Smads signalling pathway. Numerous studies (16,18,23-25) have reported that *G. acuta* exerted a protective effect against injury of the heart and the aorta of rats under various conditions, such as I/R. However, the effect and specific mechanism of action of *G. acuta* in cardiovascular damage and inflammation under hypercholesterolaemic conditions remain unclear. The aim of the present study was to explore the potential role of *G. acuta* in mitigating cardiovascular damage and inflammation in diet-induced hypercholesterolaemic rats.

## Materials and methods

### 

#### Collection and preparation of plant materials

*G. acuta* was purchased from The Darhan Muminggan Joint Banner mongolian medicine plantation, Hulunbeier district of Inner Mongolia and was identified and authenticated by Professor Yu-Ping Yan in the field of medicinal plants (College of Pharmacy, Hebei University of Chinese Medicine, Shijiazhuang, China). The plants were air-dried and then chopped. *G. acuta* (64.51 g) was soaked in 1,400 ml 25˚C distilled water for 30 min. The mixture was boiled in two batches and combined twice with the filtrate. The mixture was used at a quantity of 537 ml to obtain a suspension of *G. acuta* with a concentration of 0.12 g/ml.

#### Animals and experimental design

The Ethics Committee of Hebei University of Chinese Medicine (Shijiazhuang, China) approved and supervised the present study (approval no. DWLL2018016). A total of 32 specific-pathogen free male Sprague-Dawley (SD) rats, aged 6-7 weeks, weighing 160-180 g, were purchased from Beijing Vital River Laboratory Animal Technology Co., Ltd. (license no. SCXK 2016-0006) and all rats had free access to food and water. They were kept at room temperature with 60% humidity and 12-h light/dark cycle. After 1-week adaptive feeding, the rats were randomized into four groups (8) as follows: i) Control group (Control); ii) control administration group (Control-G), iii) model group (Model); and iv) model administration group (Model-G). While animals in the Model and Model-G groups received a high-fat diet (high-fat feed ratio, 80.4% basic feed + 2% cholesterol + 10% lard + 0.5% sodium cholate + 0.1% propylthiouracil + 5% sugar + 2% yolk powder) to induce preliminary hypercholesterolaemia, Control and Control-G animals received normal feed (100% basic feed: 248.48 g/kg crude protein + 65.18 g/kg crude fat). Normal and high-fat feed were provided and prepared by Hebei Medical University (Shijiazhuang, China). On the basis of the previous study, a 1.2 g/kg *G. acuta* dosage solution was designed (26). After the third week of modelling, the rats of the Control-G and Model-G groups were administered water extract of *G. acuta* and the other groups were treated with the same 10 ml/kg of physiological saline for 2 weeks.

At the end of the experiment, all rats were only administered water for the final 12 h. All rats were anesthetized with 50 mg/kg pentobarbital sodium (Merck KGaA) and euthanized using cervical dislocation. Following anaesthesia, blood was collected from the inferior vena cava for analysis of blood indicators. The serum was separated by centrifugation at 12,000 x g for 15 min at 4˚C and stored in a refrigerator at -80˚C for further analysis. The heart was weighed and fixed with the thoracic aorta in 10% (v/v) formalin 24 h at room temperature for histopathological studies, and the rest of heart and thoracic aorta were stored at -80˚C.

#### Blood biochemical index test

The serum levels of total cholesterol (TC; cat. no. OSR6216; Beckman Coulter, Inc.), triglycerides (TG; cat. no. OSR61118; Beckman Coulter, Inc.), low-density lipoprotein (LDL; cat. no. A113-1-1; Nanjing Jiancheng Bioengineering Institute), high-density lipoprotein (HDL; cat. no. A112-1-1; Nanjing Jiancheng Bioengineering Institute), lactate dehydrogenase (LDH; cat. no. A020-1-2; Nanjing Jiancheng Bioengineering Institute), creatine kinase (CK; cat. no. A032-1-1; Nanjing Jiancheng Bioengineering Institute), tumour necrosis factor-α (TNF-α; cat. no. SXR063; Shanghai Senxiong Biotech Industry, Co., Ltd.) and interleukin-10 (IL-10; cat. no. SXR035; Shanghai Senxiong Biotech Industry, Co., Ltd.) were assessed strictly according to the manufacturer's instructions.

#### Histopathological examination of the heart and thoracic aorta

The heart and thoracic aortas isolated from each group were fixed in 10% (v/v) formalin in 50 mm potassium phosphate buffer (pH 7.0) for 24 h at 4˚C. The tissues were subsequently embedded in paraffin, cut into 4-µm sections, and stained 5 min at room temperature with hematoxylin and then 1 min with eosin at room temperature. The sections were observed and images were captured using a light microscope with a Leica DFC 320 digital camera (magnification, x400; Leica Microsystems, Inc.).

#### Immunohistochemical analysis of IKKβ, p-IKKβ, IκBα and p-IκBα in the heart and thoracic aorta

Each section was dewaxed with a dimethylbenzene gradient and dehydrated using an alcohol gradient. The sections were then incubated with 3% H_2_O_2_ for 15 min in the dark, blocked with 100% goat serum for 20 min at room temperature (cat. no. ZLI-9056; ZSGB-BIO; OriGene Technologies, Inc.), and then rinsed three times with PBS. The primary antibodies [IKKβ (1:100; cat. no. A2087; ABclonal Biotech Co., Ltd.), p-IKKβ (1:400; cat. no. bs-5398R), IκBα (1:800; cat. no. bsm-33441M) and p-IκBα (1:200; cat. no. bs-5515R; all from BIOSS)] were incubated with the sections at 4˚C overnight. Next, rabbit two-step HRP-secondary antibody polymers (cat. no. PV-6001; ZSGB-BIO; OriGene Technologies, Inc.) were added for 60 min at room temperature and then the avidin-biotin-peroxidase complex (cat. no. PK-6200; Vector Laboratories, Inc.; Maravai LifeSciences) was added for 120 min at room temperature. The sections were stained with diaminobenzidine (DAB) reagent 10 min at room temperature, dehydrated with alcohol gradient and DAB and finally mounted using neutral balsam. The sections were viewed under a light microscope (magnification, x400) and analyzed using ImageJ software (v. d1.47; National Institutes of Health).

#### Western blot analysis for p-IKKβ, p-IκBα and p-NF-κB in the heart and p-NF-κB in the thoracic aorta

The protein extract from the frozen tissues of the heart and thoracic aorta were determined using a BCA Protein Assay Kit (cat. no. P0010; Beyotime Institute of Biotechnology) to ensure 20 µg protein per lane and were separated by 10% SDS-PAGE and then transferred onto polyvinylidene difluoride membranes. Membranes were blocked 4˚C for 5 h with 5% non-fat dry milk in Tris-buffered saline with 0.05% Tween-20 and left overnight. The blots were incubated with primary antibodies for GAPDH (1:1,000; cat. no. bs-0755R; BIOSS), IKKβ (1:100; cat. no. AF6013; Affinity Biosciences), p-IKKβ (1:400; cat. no. bs-5398R), IκBα (1:800; cat. no. bsm-33441M), p-IκBα (1:200; cat. no. bs-5515R; all from BIOSS), NF-κB (1:1,000; product no. 8242; Cell Signaling Technology, Inc.), p-NF-κB (1:250; cat. no. ab247871; Abcam) overnight at 4˚C and then incubated with a secondary antibody (1:10,000; cat. no. ZB2301; ZSGB-BIO; OriGene Technologies, Inc.) conjugated to horseradish peroxidase (1:6,500; Biosharp Life Sciences) for 2 h at room temperature. After the treatment of Super ECL Detection Reagent (cat. no. 36208ES60; Shanghai Yeasen Biotechnology Co., Ltd.), the protein bands were quantified by transmittance densitometry using ImageJ software (v. d1.47; National Institutes of Health). The relative protein band intensity was expressed as the ratio of each protein to the reference GAPDH.

#### Statistical analysis

All statistical analyses were completed using SPSS 22.0 software (IBM Corp). The data are presented as the mean ± SD. Differences among the four groups were assessed using one-way analysis of variance followed by Tukey's post hoc test. P<0.05 was considered to indicate a statistically significant difference.

## Results

### 

#### Effects of G. acuta on serum lipids

**Figure 1:**
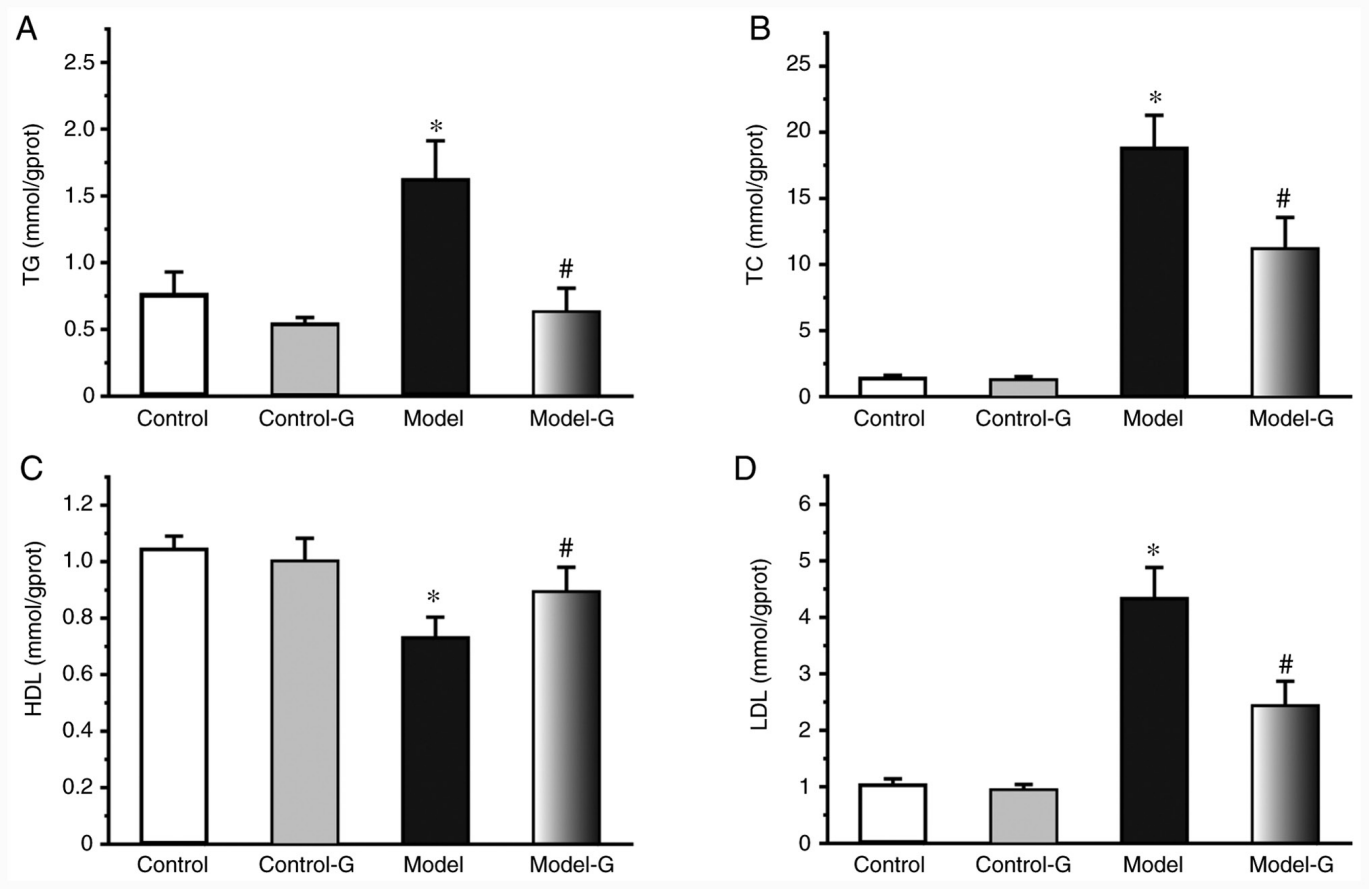
Effects of *G. acuta* on serum lipid levels. Serum (A) TG, (B) TC, (C) HDL and (D) LDL levels following treatment with *G. acuta*. Data are presented as the mean ± SD. ^*^P<0.05 vs. the Control group; ^#^P<0.05 vs. the Model group (n=8 per group). *G. acuta*, *Gentianella acuta*; TG, triglycerides; TC, total cholesterol; HDL, high-density lipoprotein; LDL, low-density lipoprotein.

Compared with the Control group, the TG, TC and LDL levels of the Model and Model-G groups were significantly increased, while the HDL level was significantly decreased (P<0.05). In addition, the levels of TG, TC and LDL were significantly decreased, and those of HDL were significantly increased in the Model-G group compared with those in the Model group (Fig. 1; P<0.05).

#### Effects of G. acuta on IL-10 and TNF-α in the serum

**Figure 2:**
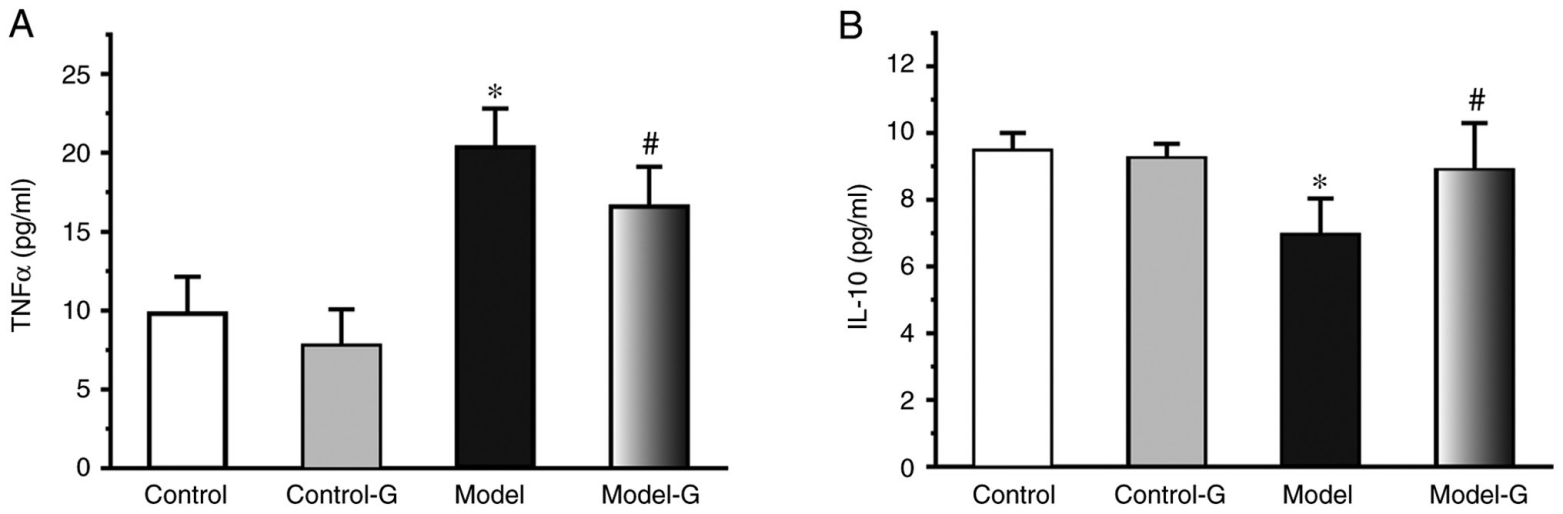
Effects of *G. acuta* on TNF-α and IL-10 levels in the serum. Serum (A) TNF-α and (B) IL-10 levels following treatment with *G. acuta*. Data are presented as the mean ± SD. ^*^P<0.05 vs. the Control group; ^#^P<0.05 vs. the Model group (n=8 per group). *G. acuta*, *Gentianella acuta*; TNF-α, tumour necrosis factor-α; IL-10, interleukin-10.

Compared with the Control group, the TNF-α levels in the Model group were increased >2-fold (P<0.05). Compared with the Model group, TNF-α was significantly decreased in the Model-G group (P<0.05). The IL-10 levels in the Model group were significantly decreased compared with those in the Control and Model-G groups (Fig. 2; P>0.05).

#### Effects of G. acuta on CK and LDH in the serum

**Figure 3:**
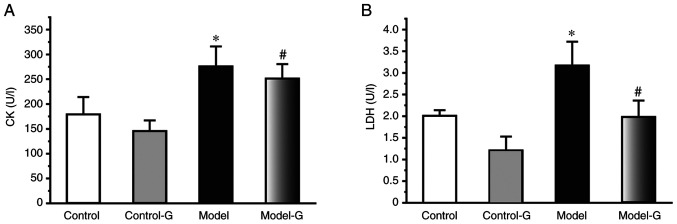
Effects of *G. acuta* on CK and LDH levels in the serum. Serum (A) CK and (B) LDH levels following treatment with *G. acuta*. Data are presented as the mean ± SD. ^*^P<0.05 vs. the Control; ^#^P<0.05 vs. the Model group (n=8 per group). *G. acuta*, *Gentianella acuta*; CK, creatine kinase; LDH, lactate dehydrogenase.

The CK and LDH levels of the Model group were significantly higher compared with those of the Control and Model-G groups (P<0.05). Compared with the Model group, the level of CK and LDH were significantly decreased in the Model-G group (P<0.05; Fig. 3).

#### Effects of G. acuta on morphological and histological changes in the heart

**Figure 4:**
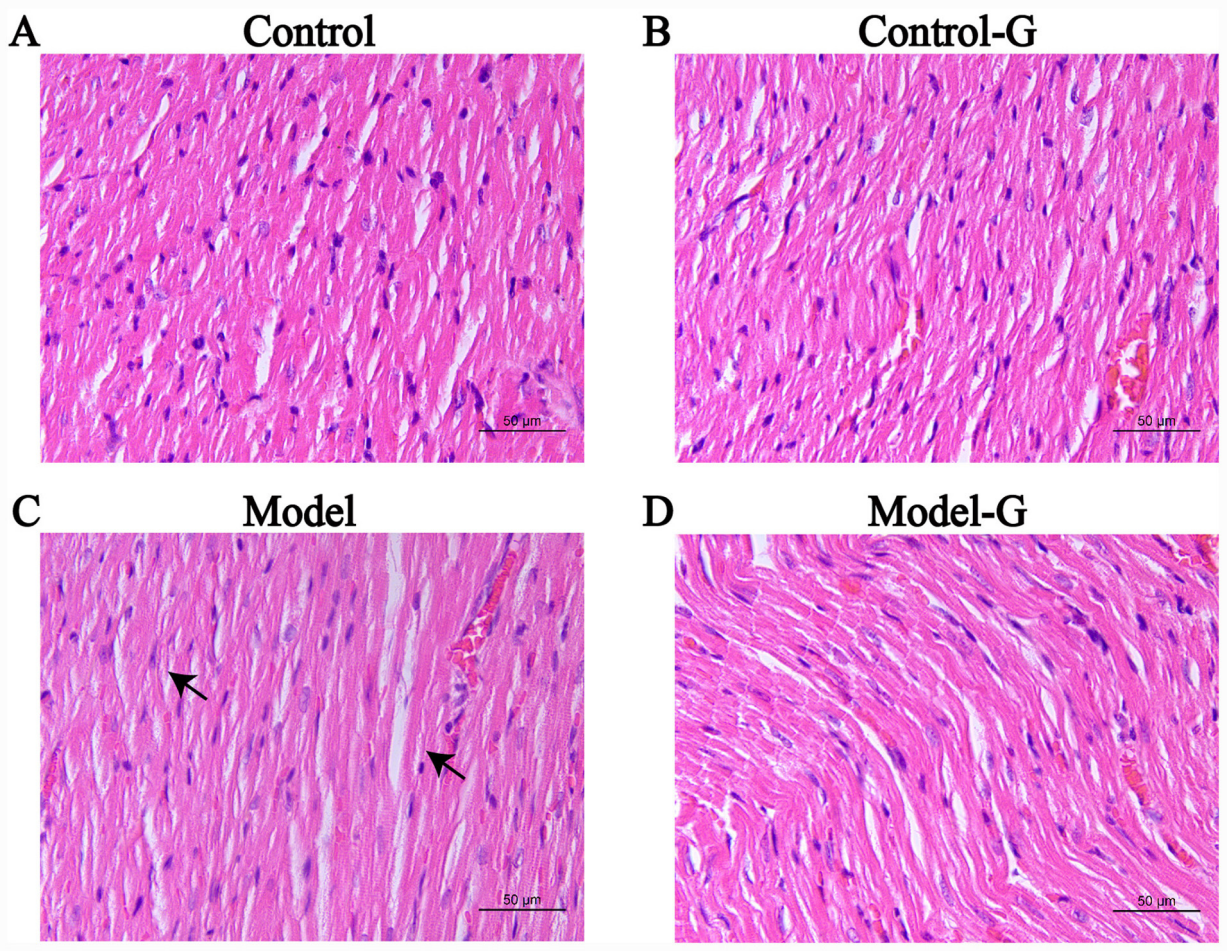
Effects of *G. acuta* on the histopathological changes in the heart. Heart tissues obtained from the (A) Control, (B) Control-G, (C) Model and (D) Model-G groups. The arrows indicated histopathological changes in the Model group. Scale bar, 50 µm. *G. acuta*, *Gentianella acuta*.

The cardiomyocytes in the Control group were arranged in an orderly manner with uniform nuclei, uniform H&E staining of the cytoplasm and obvious striations. The arrangement of cardiomyocytes in the Model group was disordered, with burrs, slightly blurry striations, and certain sections were revealed to have lipid droplets. The arrangement of cardiomyocytes in the Model-G group was improved and appeared orderly (Fig. 4).

#### Effect of G. acuta on IKK/IκB/NF-κB in the heart

**Figure 5:**
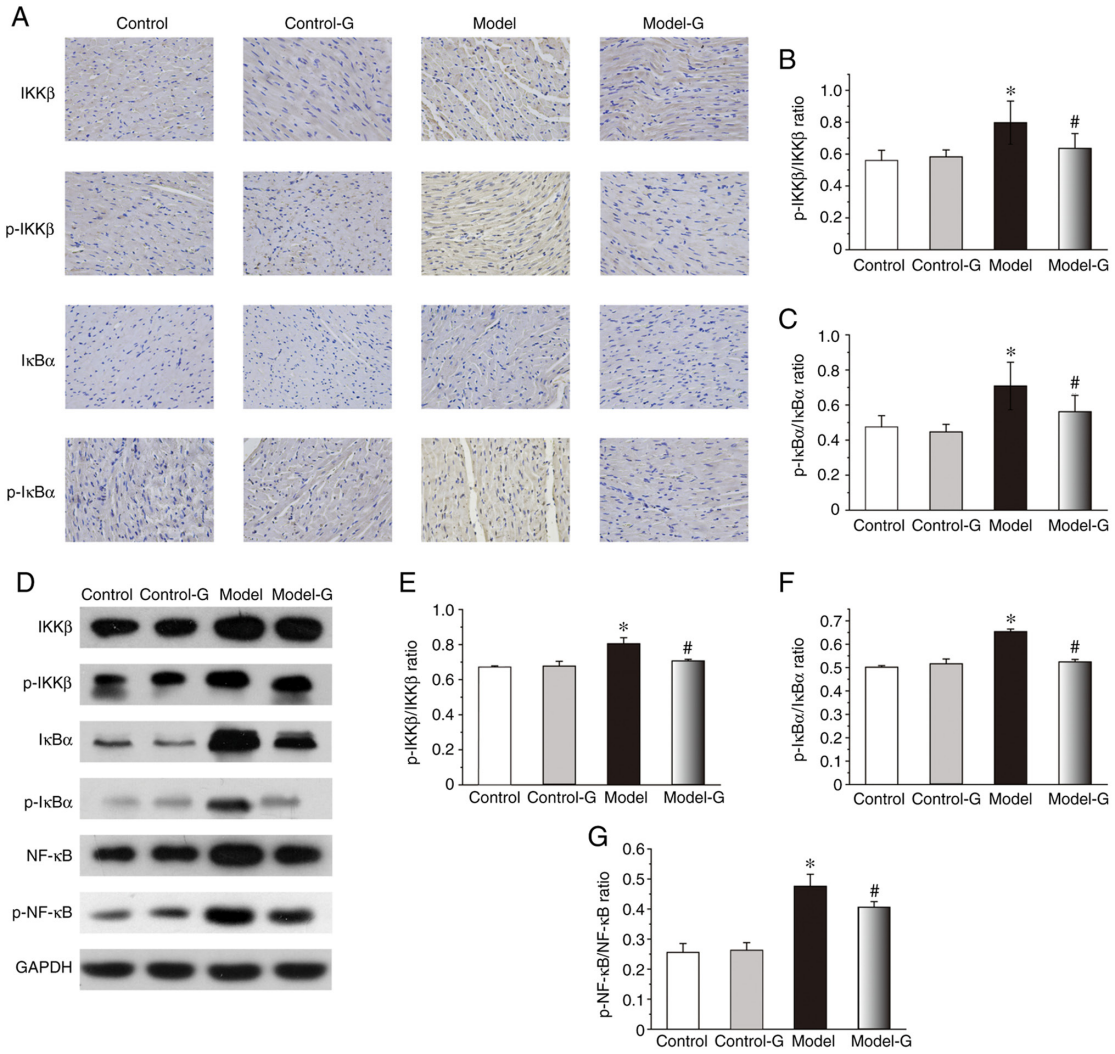
Effects of *G. acuta* on p-IKKβ, IKKβ, p-IκBα, IκBα and p-NF-κB expression levels in the heart. (A) Immunohistochemical staining for p-IKKβ, IKKβ, p-IκBα and IκBα in the heart (magnification, x400). Heart tissues were obtained from the Control, Control-G, Model and Model-G groups. (B) Expression of p-IKKβ/IKKβ in the heart. (C) Expression of p-IκBα/IκBα in the heart. (D) Typical western blot bands. (E) Expression of p-IKKβ/IKKβ in the heart was quantified by densitometry. (F) Expression of p-IκBα/IκBα in the heart was quantified by densitometry. (G) Expression of p-NF-κB/NF-κB in the heart was quantified by densitometry. Scale bar, 200 µm. Data are presented as the mean ± SD. ^*^P<0.05 vs. the Control group and ^#^P<0.05 vs. the Model group (Immunohistochemistry: n=8 per group; western blot: n=3 per group). *G. acuta*, *Gentianella acuta*; p-, phosphorylated; IKKβ, inhibitor of NF-κB kinase β; IκBα, inhibitor of NF-κB α; NF-κB, nuclear factor κB.

The IKKβ, p-IKKβ and p-IκBα protein expression levels of the Model group were markedly higher compared with those in the other three groups. While changes in the expression of IκBα were not significant among the four groups, the levels of p-IKKβ/IKKβ and p-IκBα/IκBα in the Model group were significantly higher than the other groups (Fig. 5A-C). Compared with the Control and Model-G groups, the protein levels of p-IKKβ/IKKβ, p-IκBα/IκBα and p-NF-κB/NF-κB in the Model group were significantly increased (Fig. 5D-G).

#### Effects of G. acuta on morphological and histological changes in the thoracic aorta

**Figure 6:**
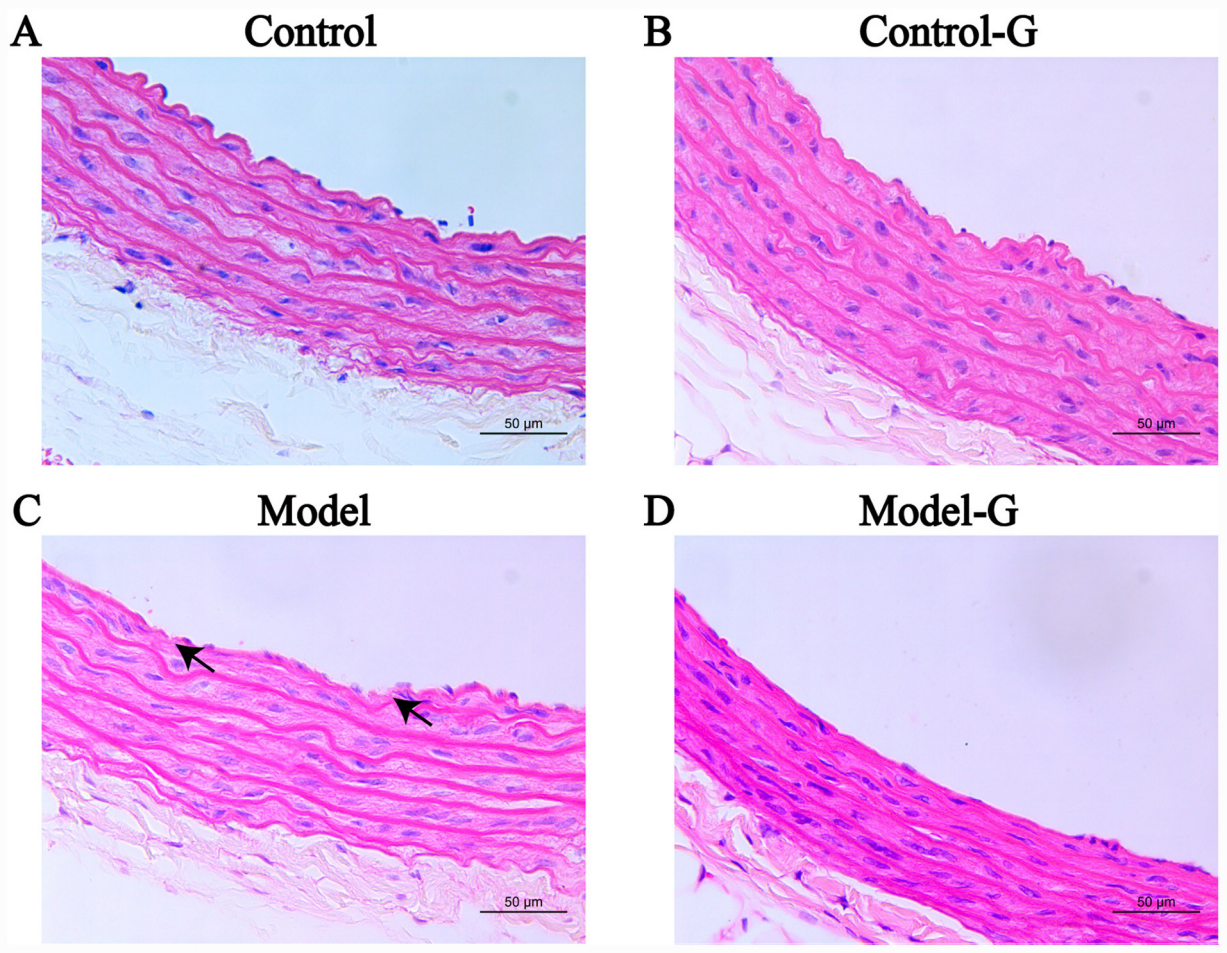
Effect of *G. acuta* on histopathological changes of the thoracic aorta. Thoracic aorta samples obtained from the (A) Control, (B) Control-G, (C) Model and (D) Model-G groups. The arrows indicate histopathological changes in the Model group. Scale bar, 50 µm. *G. acuta*, *Gentianella acuta*.

The arterial intima in the Control group was relatively smooth, with clear boundaries between the intima, media and adventitia, and endothelial cell layer continuity. The intima of the arteries of the Model group was uneven, and some endothelial cells had lost their continuity. Intimal concavity was improved in the Model-G group. The intima of the Control-G group was damaged (Fig. 6).

#### Effect of G. acuta on IKK/IκB/NF-κB in the thoracic aorta

**Figure 7:**
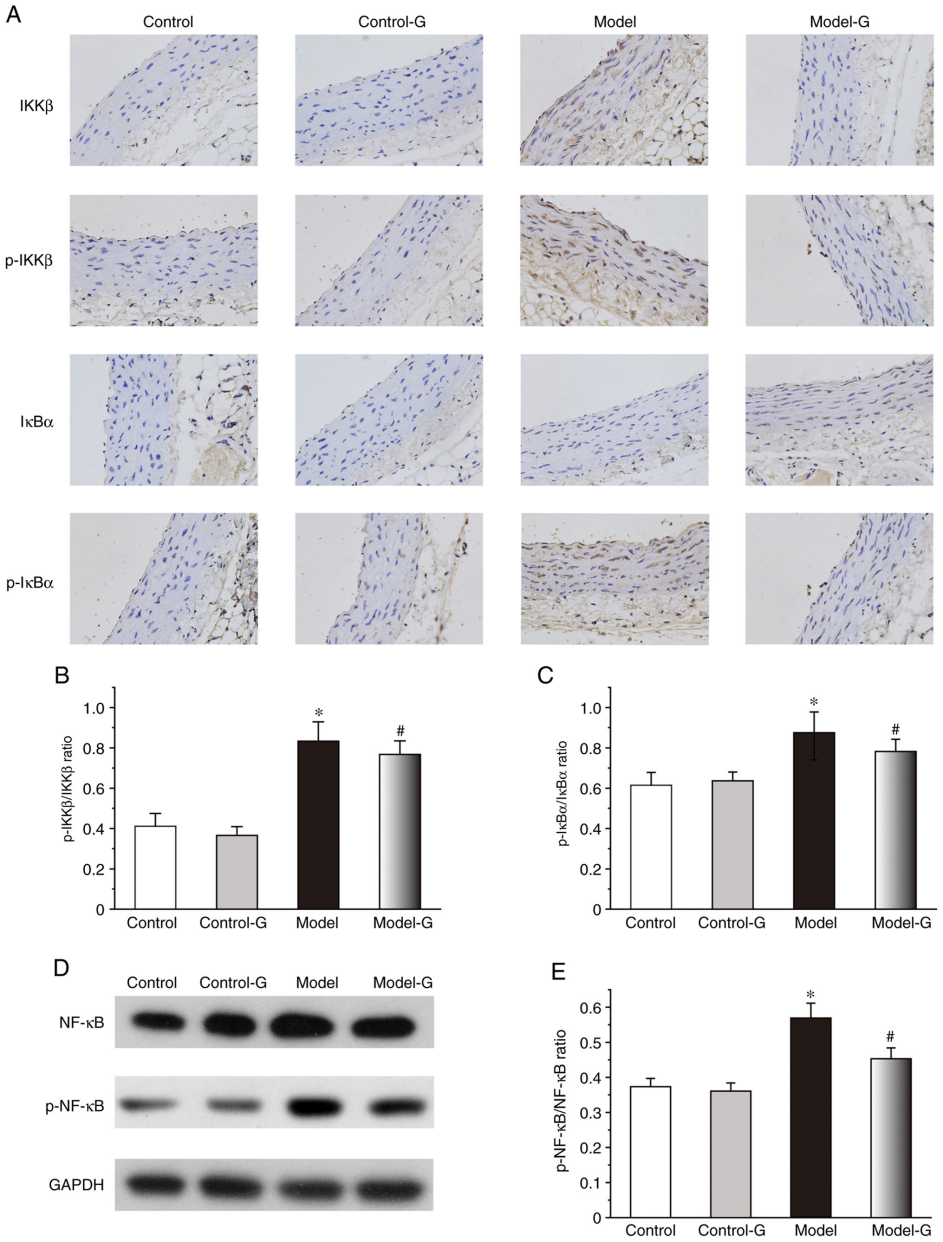
Effects of *G. acuta* treatment on p-IKKβ, IKKβ, p-IκBα, IκBα and p-NF-κB expression levels in the thoracic aorta. (A) Immunohistochemical staining for p-IKKβ, IKKβ, p-IκBα and IκBα in the thoracic aorta (magnification, x400). Thoracic aorta samples were obtained from the Control, Control-G, Model and Model-G groups. (B) Expression of p-IKKβ/IKKβ in the thoracic aorta. (C) Expression of p-IκBα/IκBα in the thoracic aorta. (D) Typical western blot bands. (E) Expression of p-NF-κB/NF-κB in the thoracic aorta was quantified by densitometry. Scale bar, 200 µm. Data are presented as the mean ± SD. ^*^P<0.05 vs. the Control group; ^#^P<0.05 vs. the Model group (Immunohistochemical: n=8 per group; western blot: n=3 per group). *G. acuta*, *Gentianella acuta*; p-, phosphorylated; IKKβ, inhibitor of NF-κB kinase β; IκBα, inhibitor of NF-κB α; NF-κB, nuclear factor kappa-B.

The IKKβ, p-IKKβ and p-IκBα protein expression levels of the Model group were significantly higher compared with those in the other three groups. While the changes in the expression of IκBα were not significant among the four groups, the levels of p-IKKβ/IKKβ and p-IκBα/IκBα in the Model group were higher than the other three groups (Fig. 7A-C). Compared with the Model group, the levels of p-NF-κB/NF-κB in the Control and Model-G groups were significantly decreased (Fig. 7D and E).

## Discussion

Several studies have reported that hypercholesterolaemia is not only a risk factor for AS development, but also an important cause of the exacerbation of AS (27,28). It has been revealed that *G. acuta* exerts a protective effect against myocardial ischemia (19). On this basis, its anti-AS effects and mechanism were studied herein. In the present study, a rat model of hypercholesterolaemia was established using a high-fat diet (29) to explore the effect and mechanisms of *G. acuta* in mitigating cardiovascular damage and inflammation.

Hypercholesterolaemia model rats exhibited increases in serum lipids and inflammatory factors, aortic muscular layer thickening and widespread myocardial structural disruption. These histopathological changes in the body were important formative indices of hypercholesterolaemia, with some beneficial changes appearing in the Model-G group, such as improved aortic wall structure and neatly arranged myocardial cells. Lipid deposition has been identified as an important cause of AS, which can lead to the increase of free radicals, thereby damaging endothelial cell function (30-32). Thus, the release of protective factors is reduced, leading to a reduction in the tightness of endothelial cells and increased permeability, which in turn results in increased lipid deposition, forming a vicious circle (33). As a consequence of a continuous high-fat diet, LDL is elevated and deposited in the endothelial cells of the arteries in which it is oxidized to ox-LDL, which can cause necrosis and disintegration of macrophages, release of lipids from atheromatous necrosis and plaque formation (34). When comparing the Model-G and the Model groups, it was revealed that *G. acuta* could effectively reduce the serum lipid level with further increasing the level of HDL.

A change in TNF-α and IL-10 levels in the serum of hypercholesterolaemic rats was also observed. TNF-α and IL-10 are important inflammatory factors leading to AS. TNF-α has been reported to promote the production of various inflammatory cytokines through T cells and has been identified as an important indicator of inflammation (35). Conversely, IL-10 has been reported to inhibit mononuclear macrophages from performing specific immune functions, such as the release of inflammatory mediators (36). Compared with the Control group, the levels of TNF-α were significantly increased, and those of IL-10 were decreased in the Model group. Following treatment with *G. acuta*, TNF-α and IL-10 levels were significantly altered in the Model group. These results demonstrated that the inflammatory response induced by the high-fat diet was inhibited by *G. acuta.*

CK and LDH have been revealed to be important indices reflecting functional heart status (37). Compared with the control group, the levels of CK and LDH in the blood vessels of the Model group were significantly decreased, indicating the protective effect of *G. acuta* in the heart. It was also revealed by H&E staining that the arrangement of cardiomyocytes in the Model-G group was improved and appeared orderly. These results further demonstrated that *G. acuta* effectively alleviated cardiovascular damage and inflammation in diet-induced hypercholesterolaemic rats.

NF-κB has been identified as an important nuclear factor that controls inflammatory cytokines and is normally bound to IκB in the cytoplasm. After NF-κB has been activated and translocated to the nucleus, several downstream inflammation-related factors, such as TNF-α and IL-6, promote its synthesis and release. Such factors are important causes of the occurrence and deterioration of AS (38-40). As a pattern recognition receptor, activated IKK is a major upstream target for NF-κB regulation. Multiple members of the IKK family, such as IKKα and IKKβ, have important regulatory effects on the activity of NF-κB. Both of these have been revealed to phosphorylate the IκB protein at different serine residues, while the main function has been assumed by IKKβ (41). Thus, IKKβ is an important indicator of NF-κB activation. When *G. acuta* was administered, the expression of p-IKKβ/IKKβ and p-IκBα/IκBα both in the heart and thoracic aorta in the Model-G group were significantly decreased. Compared with the Model group, the phosphorylation ratio of IKKβ, IκBα, NF-κB in the heart and NF-κB in thoracic aorta was decreased in the Model-G group. In addition, *G. acuta* significantly decreased the expression levels of IKKβ, p-IKKβ and p-IκBα in endothelial cells of the thoracic aorta, indicating its protective role. These results demonstrated that the anti-inflammatory effect of *G. acuta* may be mediated by inhibiting the IKKβ/IκBα/NF-κB pathway in the heart as well as the thoracic aorta.

In conclusion, *G. acuta* mitigated cardiovascular damage and inflammation in diet-induced hypercholesterolaemic rats, possibly through the inhibition of the IKK-β/IκB/NF-κB pathway. Thus, *G. acuta* may prove useful in the treatment of hypercholesterolaemia.

A limitation of the present study was that it lacked direct assessment of physiological parameters and immunohistochemical analysis could reveal the expression levels but was weaker in protein comparison than western blotting in thoracic aorta. In addition, *G. acuta* water extract was selected, but water extract is comprised of numerous components and these were not fractionated and explored individually.

## Data Availability

The datasets used and/or analyzed during the current study are available from the corresponding author on reasonable request.
